# Oncogenic and tumor suppressor function of MEIS and associated factors

**DOI:** 10.3906/biy-2006-25

**Published:** 2020-12-14

**Authors:** Birkan GİRGİN, Medine KARADAĞ-ALPASLAN, Fatih KOCABAŞ

**Affiliations:** 1 Regenerative Biology Research Laboratory, Department of Genetics and Bioengineering, Faculty of Engineering, Yeditepe University, İstanbul Turkey; 2 Graduate School of Natural and Applied Sciences, Yeditepe University, İstanbul Turkey; 3 Meinox Pharma Technologies, İstanbul Turkey; 4 Department of Medical Genetics, Faculty of Medicine, Ondokuz Mayıs University, Samsun Turkey

**Keywords:** MEIS, cancer, small molecules, MEIS inhibitors

## Abstract

MEIS proteins are historically associated with tumorigenesis, metastasis, and invasion in cancer. MEIS and associated PBX-HOX proteins may act as tumor suppressors or oncogenes in different cellular settings. Their expressions tend to be misregulated in various cancers. Bioinformatic analyses have suggested their upregulation in leukemia/lymphoma, thymoma, pancreas, glioma, and glioblastoma, and downregulation in cervical, uterine, rectum, and colon cancers. However, every cancer type includes, at least, a subtype with high MEIS expression. In addition, studies have highlighted that MEIS proteins and associated factors may function as diagnostic or therapeutic biomarkers for various diseases. Herein, MEIS proteins and associated factors in tumorigenesis are discussed with recent discoveries in addition to how they could be modulated by noncoding RNAs or newly developed small-molecule MEIS inhibitors.

## 1. Introduction

Homeobox (Hox) genes are transcription factors that are characterized by a conserved 60-amino acid DNA-binding domain called homeodomain (HD) (Abate-Shen, 2002). They are important regulators of cell fate, development, tumorigenesis, and stem cell function (Abate-Shen, 2002). They finely regulate organogenesis in nonvertebrates and are also involved in molecular pathways that determine vertebrate development (Mallo and Alonso, 2013). The expression of Hox genes is modulated by nuclear dynamics, transcriptional regulation, long noncoding RNAs (lncRNAs), RNA processing, miRNAs, and translational events (Mallo and Alonso, 2013).

The 3 amino acid loop extension (TALE) proteins are one of the major HD proteins, that include 27 members characterized by Meis1-3, Pbx1-4, Irx1-6, Mkx, Pknox1-2, Tgif1-2, and their pseudogenes (Sitwala et al., 2008; Pillay et al., 2010). There are 3 Meis isoforms, called Meis1, Meis2, and Meis3, in mammals (Sonnet et al., 2012). Interestingly, expression levels of Meis isoforms are quite different from one another in each cell (Sonnet et al., 2012). The Meis1 gene was first identified in myeloid leukemia using BXH2 mice. It acts as a viral integration site in myeloid leukemia mice (Moskow et al., 1995).

MEIS1 and PBX regulatory protein-1 (PREP1) compete functionally with each other (Blasi et al., 2017). Meis1 overexpression induces tumor formation in Prep1-silenced mouse embryonic fibroblasts (Dardaei et al., 2014). MEIS, HOX, and PBX proteins bind to DNA as a heterodimer or trimeric structures (Chang et al., 1997). MEIS-, HOX-, and PBX-generated trimeric structures increase the half-life of the complex (Shanmugam et al., 1999). PBX proteins modulate cellular signaling pathways, including Notch and Smad, and are involved in chromosomal remodeling (Laurent et al., 2008). When PBX transcription factors malfunction, organ failure, and cardiovascular diseases could occur during development (Laurent et al., 2008). On the other hand, the deletion of Meis1 downregulates p21, p15, p16, and p19arf expression in cardiomyocytes and improves the cell cycle (Muralidhar and Sadek, 2016). Intriguingly, the overexpression of MEIS proteins may cause caspase-dependent apoptosis in some cells (Wermuth and Buchberg, 2005). PBX-MEIS1 protein-protein interaction is required for the induction of caspase-3- and caspase-8-dependent apoptosis (Wermuth and Buchberg 2005). MEIS2-PBX heterodimer finely regulates pancreatic and duodenal homeobox 1 (Pdx1) expression in the acinar cells of the pancreas (Swift et al., 1998). Generally, Meis3 expression is involved in neural development (Uribe and Bronner, 2015). MEIS3 provides β-cell survival by targeting phosphoinositide-dependent protein kinase 1 (Liu et al., 2010). However, HOXA9-MEIS1 interaction has an inhibitory effect on apoptosis in Reh human lymphoblastoma cells, HL-60, 32Dcl3, and NIH3T3 cells (Wermuth and Buchberg, 2005).

Since the expression of Meis isoforms is different in various cell types and tissues, this suggests that expression of MEIS proteins may have cell-specific outcomes. Somers et al. (2016) studied cytotoxicity in different mixed-lineage leukemia (MLL) cells. They showed that the downregulation of Meis1, Hoxa9, c-Myc, and Bcl2 were associated with caspase-dependent apoptosis. The upregulation of MiR-155, which is a major inducer of the Meis pathway, leads to caspase-dependent apoptosis involving c-Jun N-terminal kinase signaling in acute myeloid leukemia (AML) (Palma et al., 2014). In addition, transient overexpression of Meis1 induces caspase-dependent apoptosis in Reh human lymphoblastoma, Jurkat T, HL-60, and 32Dcl3 cells (Wermuth and Buchberg, 2005). Although the MEIS-dependent apoptosis response has not yet been fully elucidated, there is a relationship between MEIS proteins and apoptosis.

MEIS proteins have been studied in developmental biology, stem, and progenitor cells in different cell types and conditions. Using clustered regularly interspaced short palindromic repeats/Cas9 technology, it was revealed that Meis1 is a significant regulator for the differentiation of human pluripotent stem cells (hPSCs) into functional hematopoietic cells (Wang et al., 2018a). Meis1 is essential for normal hematopoiesis, as was indicated by Meis1 mutant mice having an internal hemorrhage, liver hypoplasia, and anemia (Azcoitia et al., 2005). MEIS1 suppresses the generation of reactive oxygen species (ROS) and induces scavenging by targeting hypoxia inducible factors (HIF-1α and HIF-2α) in hematopoietic stem cells (Kocabas et al., 2012). Thus, MEIS1 protects hematopoietic stem cells against ROS by maintaining the glycolytic metabolic phenotype (Kocabas et al., 2012). MEIS-PBX heterodimers are indispensable in neurogenesis (Azcoitia et al., 2005). Dopaminergic periglomerular neuron proliferation in the olfactory bulb is provided by MEIS2 via forming a heterodimer with PAX6 and DLX2 (Agoston et al., 2014). The MEIS2-PBX1 heterodimer could also alter the structure of chromatin, and thus modulate the activity of the genes. The MEIS2-PBX1 heterodimer, for instance, recruits poly-ADP-ribose (PAR) polymerase 1 (PARP1/ARTD1) in order to regulate PARP1 activity on the chromosome (Hau et al., 2017).

## 2. MEIS and associated factors in tumorigenesis

Studies have shown that MEIS proteins and their cofactors are misregulated in various cancers. These studies have highlighted that MEIS proteins maybe diagnostic and therapeutic biomarkers for cancer and other associated diseases. Thus, herein, the function of MEIS and its associated factors in solid and nonsolid tumors, as well as recent therapeutic approaches, including small molecules and noncoding RNAs in MEIS-related cancers, will be discussed.

### 2.1. Bladder cancer

Even though it is known that MEIS partners directly regulate tumor progression along with HOX proteins, the definitive role of MEIS proteins remains unclear in bladder cancer. Hoxa13 and Hoxb13 are, for instance, known to have high expression in urinary bladder cancer (Marra et al., 2013; Hu et al., 2017) Hox antisense intergenic RNA (HOTAIR) has been proposed as a marker in bladder cancer (Martinez-Fernandez et al., 2015; Berrondo et al., 2016). HOTAIR downregulates the level of microRNA-205, thereby disrupting the balance of H3K4me3 activity in bladder cancer (Sun et al., 2015). Under normal circumstances, 11 Hox paralogs in locus C are involved in healthy urogenital development; however, in the case of bladder cancer, these gene family variants are upregulated (Cantile et al., 2003).

Genetic and epigenetic modifications have an important function in carcinoma formation. DNA hypermethylation is among the most common and characterized epigenetic modification in human malignancies. The methylation level is different between low/intermediate and high-grade nonmuscle invasive bladder cancer (HG-NMIBC) cohorts for Hoxa9 and Isl1 genes, and the methylation level of the Hoxa9 promoter is significantly reduced in HG-NMIBC (Kitchen et al., 2015). Moreover, in recurrent and progressive tumor samples, Isl1 and Hoxa9 are displayed by a remarkably high level of methylation when compared to nonrecurrent tumor samples (Kitchen et al., 2015). Therefore, parallel methylation of Hoxa9/Isl1 in HG-NMIBC could be used as a predicted value for tumor recurrence and progression (Kitchen et al., 2015).

Methylation analysis performed with bladder cancer tissue samples revealed that Meis1 methylation was similar in all of the samples, regardless of the age of the patients (Beukers et al., 2013). On the other hand, Meis1 methylation analysis of patient urine samples of painless hematuria also showed that Meis1 was a significant predictor for the presence of urothelial cell carcinoma (Beukers et al., 2013). Therefore, methylation analysis itself is not sufficient for predicting Meis1 function in bladder cancer, and further research is needed.

Alternative splicing has a significant function in the posttranscriptional regulation of genes, as well as cancer development or progression. Polypyrimidine tract binding protein 1, is a protein that has a function bladder cancer metastasis, and controls Meis2 and pyruvate kinase alternative splicing via direct binding to specific introns of these mRNA transcripts, contributing to bladder cancer development (Xie et al., 2019). Meis2 was also reported as overexpressed in bladder cancer tissue when compared with normal adjacent tissue (Xie et al., 2019). Moreover, Meis2 knockdown significantly inhibits the migration and invasion capacities of bladder cancer cells (Xie et al., 2019).

Clarifying the potential role of MEIS proteins and how they are related to HOX proteins in bladder cancer will be an important step in determining the molecular mechanism of bladder cancer and developing new therapeutic approaches.

### 2.2. Breast cancer

Breast cancer is a hormone-dependent malignant cancer. However, estrogen receptor products, estrogen α, estrogen β, and progesterone, are known to also be activated in breast cancer via estrogen-independent pathways (Dudek and Picard, 2008). Therefore, a possible relationship between MEIS and TALE family proteins in breast cancer needs to be investigated in both estrogen-independent and -dependent pathways (Dudek and Picard, 2008; Zhang et al., 2013). In breast cancer, estrogen receptor induces Meis1 and Forkhead box P3 (Foxp3) upregulation (Zhang et al., 2013). MEIS1-FOXP3 interaction with a positive feed-back mechanism could enhance the expression of cancer-associated genes under the estrogen receptor pathway (Zhang et al., 2013). The expression of Meis1, along with transmembrane protein 25 (Tmem25) and Reps2, has been suggested to be a marker for breast cancer prognosis (Doolan et al., 2009). High expression of Meis1, Tmem25, and Reps2 is a determining factor for evaluating the probability of relapse and survival of breast cancer (Doolan et al., 2009). Mutations that affect HOX/PBX/MEIS interactions may also contribute to breast cancer (Dard et al., 2018). Interestingly, interactions of HOX/PBX proteins are unstable in the absence of MEIS and their expressions are aberrant in breast cancer (Dard et al., 2018). Taken together, these data indicate that MEIS1 and associated factors may demonstrate an oncogenic function in breast cancer development and progression.

### 2.3. Colorectal and gastric cancers

MEIS, HOX, and HIF-MEIS target genes are largely misregulated in colorectal cancer (Yuan et al., 2017). Distant metastasis and associated mortality are closely related to Meis2 expression in colorectal cancer (Wan et al., 2019). In vitro and in vivo studies have suggested that MEIS2 may inhibit migration, invasion, and the epithelial to mesenchymal transition of colorectal cancer (Wan et al., 2019). Moreover, high Meis2 expression may reduce the overall survival period of patients with colorectal cancer (Wan et al., 2019).

Different HOX proteins have been reported to serve for tumor formation, metastasis, lymph node metastasis, and cancer stem cells (CSCs) self-renewal in colorectal cancer (Kanai et al., 2010). PBX3 protein, which forms a heterodimer with MEIS proteins, is overexpressed in colorectal cancers (Han et al., 2014). PBX3 protein, in particular, contributes to cell proliferation, invasion, and metastasis in colorectal cancers by modulating mitogen-activated protein kinases (MAPK)/extracellular-signal-regulated kinase (ERK) signaling pathways (Han et al., 2014). The expression pattern of Hoxa9 has been found to be more characteristic of colonic adenocarcinomas (Redline et al., 1994). Hoxa9 mRNA and protein expression were reported as increased in colorectal tumor tissues when compared with normal tissues and elevated Hoxa9 expression was correlated with lymph node metastasis (Watanabe et al., 2018). In addition, the knockdown of Hoxa9 and Hoxa4 in HT29 cells leads to a significant decrease in cell proliferation, and their overexpression causes an increase in the self-renewal ability of colorectal CSCs (Bhatlekar et al., 2018). Moreover, in colorectal cancer cells, nucleus accumbens-associated protein 1 (Nac1) expression is elevated, which leads to an increase in drug resistance by upregulating Hoxa9 expression (Ju et al., 2017). On the other hand, mir133b downregulates Hoxa9 expression and inhibits colorectal tumor cell proliferation and migration (Wang et al., 2017).

The expression level of Meis1D27, a truncated splicing variant of Meis1, has been reported to be decreased in primary colorectal cancer samples (Crist et al., 2011). This suggests that Meis1D27 could function as a tumor suppressor in colorectal cancer (Crist et al., 2011). Moreover, it has been shown that the BRAF V600E mutation is related to the methylation of Meis1. In tumors and colon cancer cell lines, this situation has been associated with a reduction of both the full-length Meis1 and a truncated isoform Meis1D27 transcript expression (Dihal et al., 2013).

The development and metastasis of the esophageal squamous cell carcinoma (ESCC) involve varying and complicated signaling pathways. Rad et al. (2016) showed that the expression of Meis1 has a reverse association with lymph node involvement, metastasis, and tumor staging in ESCC (Rad et al., 2016). Enhancer of zeste homolog 2 (EZH2) leads to Meis1 downregulation during ESCC progression (Rad et al., 2016). Moreover, the expression of Meis1 was found to be inversely correlated with Sox2 expression in ESCC tumor samples (Rad et al., 2016). The inverse correlation has also been shown between mastermind-like transcriptional coactivator 1 and Meis1 in ESCC patients (Abbaszadegan and Moghbeli, 2018). On the other hand, a positive correlation between Musashi RNA-binding protein 1 and Meis1 was reported during ESCC progression (Moghbeli et al., 2016).

Meis1 may function as a tumor suppressor during gastric cancer progression (Song et al., 2017). Its expression was diminished in gastric cancer tissues or cell lines; however, when it was overexpressed, the proliferation, colony formation, and anchorage-independent growth of gastric cancer cells were repressed (Song et al., 2017). In addition, the overexpression of Meis1 could lead to G1/S cell cycle arrest and cell death of gastric cancer cells (Song et al., 2017).

### 2.4. Glioblastoma, glioma, and neuroblastoma

Glioblastoma and neuroblastoma both originate from nervous system cells (de Weille, 2014). Neuroblastoma is cancer that occurs in the nervous system during embryonic development, while glioblastoma occurs in the adult brain (de Weille, 2014). HOX proteins have been highly studied in tumors of the nervous system. Studies conducted in U-118, U-138, and normal human astrocyte glioblastoma cell lines showed that Hoxc8, Hoxc10, Hoxd1, Hoxd4, Hoxd9, Hoxd10, and Hoxd13 were expressed in glioblastoma cells, while they were not detected in healthy tissue (Guo et al., 2016). Hoxc6 and Hoxc10 overexpression regulates glioblastoma cell proliferation and migration via MAPK and PI3K/protein kinase B (AKT) signaling pathways, respectively (Guan et al., 2019; Yang et al., 2019). HOXA13 promotes cancer invasion by wingless/integration (Wnt) and transforming growth factor (TGF)-β pathways (Duan et al., 2015). Intriguingly, HOXA10 allows glioblastoma cells to develop resistance to chemotherapy agents (Kim et al., 2014).

The Pbx3 mRNA and protein content is higher in glioblastoma cells. The migration and invasion of glioblastoma cells is triggered by PBX3/MEK/ERK1/2/LIN28/let-7b (Xu et al., 2018). In vivo Pbx3 suppression resulted in a reduced invasion of glioblastoma (Xu et al., 2018). However, there is still a need to establish the direct role of MEIS proteins in glioblastoma. Given the high level of Meis1-2-3 expression in glioblastoma tissues, a study examining the relationship between MEIS1-2-3 and glioblastoma is clearly needed.

Glioma, one of the most prevalent and heterogeneous tumors of the brain, is characterized by high morbidity and death rates (Weller et al., 2015). These tumors are thought to originate from the neuroglial stem or progenitor cells (Weller et al., 2015; Chen et al., 2017). Nuclear receptor SET domain-containing protein-1 (NSD1) silencing by epigenetic modification leads to Sotos syndrome, as well as nonhereditary neuroblastoma and glioma development (Berdasco et al., 2009). Hypermethylation of Nsd1 causes the upregulation of Meis1 transcript and protein due to the absence of NSD1 binding to the Meis1 promoter in neuroblastoma cells (Berdasco et al., 2009). A high level of Meis1 transcript has also been seen in the lymphoblastoid cells of patients with Sotos syndrome as a result of NSD1 silencing (Berdasco et al., 2009). Gene expression analysis of glioma and glioblastoma cells revealed that Meis2 was one of the differentially expressed genes and it could be a prognostic biomarker for glioma and glioblastoma development, in addition to with other genes, including Meox2, Pitx2, Nr2e1, and Tfap2B (Vastrad et al., 2017). miR-638 modulates Hoxa9 expression in glioma cell lines (Zheng et al., 2018). It has been shown that miR-638 expression is related to tumor size and score in glioma. The overexpression of Hoxa9 clears the tumor suppressor effects of miR-638 (Zheng et al., 2018). Moreover, cyclin D1 and c-Myc are controlled by miR-638 and Hoxa9 (Zheng et al., 2018).

The gene expression analysis of spinal cord ependymomas revealed that 105 genes were upregulated (Kim et al., 2018). Among them, Arx, Hoxc6, Hoxa9, Hoxa5, and Hoxa3 were the 5 top-ranked genes (Kim et al., 2018). Another gene expression analysis revealed that HOTAIR was overexpressed in high-grade gliomas and it was likely controlled by DNA methylation along with the coexpression of Hoxa9 (Xavier-Magalhaes et al., 2018). Therefore, these 2 genes could be a prognostic marker for high-grade gliomas. Immunohistochemical analysis in patients with clinical intramedullary spinal tumors showed that both Hoxa9 and Hoxb13 had been upregulated (Gu et al., 2017). Moreover, they were found to be involved in spinal ependymoma and myxopapillary ependymoma development, respectively (Gu et al., 2017). Taken together, each member of the HOX family of proteins has unique functions in glioma; thus, they could be useful as specific markers of the disease.

Analysis of Hox genes in neuroblastoma began in the early 1990s (Peverali et al., 1990). Neuroblastoma differentiation using retinoic acid was found to result in Hox gene upregulation (Manohar et al., 1993). The expression levels of Hoxc6, Hoxd1, and Hoxd8 genes were increased significantly by chemical induction, while the expression of Hox genes was undetectable in undifferentiated neuroblastomas. In addition, the expression of Hoxd4 and Hoxd9 were detected at low levels in human neuroblastoma cells (Manohar et al., 1996). Hoxc9 may act as a tumor suppressor and upregulated Hoxc9 may activate the intrinsic apoptosis signaling pathways in neuroblastoma cells (Kocak et al., 2013).

The TALE homeobox genes are very important for the standard development of the nervous system. They have been implicated in neuroblastoma, glioma, and glioblastoma. Most TALE subfamilies (including Meis1, Meis2, and Pbx2) are upregulated in a set of neuroblastoma cell lines, suggesting that the regulation of TALE transcription is functional in tumorigenesis (Jones et al., 2000; Spieker et al., 2001; Geerts et al., 2003; Berdasco et al., 2009). Intriguingly, stable transfection of the dominant-negative variant of MEIS1 has generated clones with altered cell proliferation, increased differentiated phenotype, and elevated contact inhibition and cell death. Similarly, MEIS2 has been shown to be essential for neuroblastoma cell survival and proliferation (Zha et al., 2014). In short, MEIS proteins may act as oncogenes in neuroblastoma, as reported by Geerts et al. (2003).

PHNOX2B transcription factor is expressed under the control of MEIS1-PBX1, AP-1, and NF-κB complexes and has been shown to be one of the major triggering factors of neuroblastoma (Di Zanni et al., 2015). It was first shown in 2001 that Meis1 was amplified and overexpressed in neuroblastoma cancer cells (Spieker et al., 2001). Spieker et al. (2001) showed, in a serial analysis of gene expression, that MEIS1 proteins were overexpressed in 22 out of 24 examined neuroblastoma cell lines (Spieker et al., 2001). The overexpression of Meis1 and Meis2 in neuroblastoma cells is associated with increased tumor progression (Geerts et al., 2003). The impairment of cellular proliferation, differentiated phenotype, and induced cellular death occur in the case of dominant-negative splice variants of Meis1 expression in neuroblastoma cells (Geerts et al., 2003). The inhibition of Meis1 and Meis2 expression also causes apoptosis in neuroblastoma cells (Geerts et al., 2003). Hence, an increased expression of Meis1 and Meis2 may be involved in the promotion of neuroblastoma formation; thus, they may act as oncogenes (Geerts et al., 2003).

### 2.5. Kidney cancer

MEIS1 suppresses cellular differentiation and induces self-renewal in embryonic kidney malignancies (Dekel et al., 2006). MEIS1 has a pivotal function in fetal liver formation, and it is rapidly downregulated during in vitro differentiation of the kidney (Dekel et al., 2006). Studies have shown that miRNA-204 expression in nephroblastomas is reversely correlated with Meis1 expression (Koller et al., 2014). While miRNA-204 is downregulated, Meis1 and its binding partner, Pbx2, are upregulated (Koller et al., 2014). In kidney cancer, HOX proteins are also aberrantly expressed (Shears et al., 2008). When the interaction between HOX proteins (such as HOXA3, HOXA4, HOXA5, HOXA6, HOXA9, HOXB4, HOXB5, HOXB7, HOXC4, HOXC9, HOXD8, HOXD9, and HOXD10) and PBX proteins are disrupted, necrosis and apoptosis may occur in renal cancer cell lines, such as CaKi-2 and 769-P (Shears et al., 2008). Studies have suggested that MEIS1 may function as a tumor suppressor in the progression of clear cell renal cell carcinoma (ccRCC), since the endogenous expression of Meis1 is reduced in ccRCC cell lines (Zhu et al., 2017). Furthermore, the elevation of MEIS1 expression significantly blocks the proliferation and apoptosis of ccRCC cells (Zhu et al., 2017).

### 2.6. Leukemia/lymphoma

Leukemia is a disorder in which the red blood cells and platelets are disabled by an aberrant increase of white blood cells (Mudgapalli et al., 2019). Meis1 has been identified as one of the primary factors in the formation of leukemia (Lasa et al., 2004). Mutations in the MEIS-HOX signaling pathway and its downstream proteins have been found to cause MLL (Zhou et al., 2014). PU.1 transcription factor cross-talks with the MEIS-HOX signaling pathway in leukemia cells, promotes cell cycle progression, and inhibits cell death (Zhou et al., 2014). In vivo studies have shown that PU.1 mutation contributes to MLL development by modulating MEIS-HOX downstream genes (Zhou et al., 2014). MEIS1-PBX and HOX-PBX heterodimer complexes have been shown to occupy promoter regions of leukemia-related genes (Wang et al., 2006). The MLL1- WD repeat-containing protein 5 (WDR5) protein complex allows high expression of Meis1 and Hox by methylation activity on DNA (Karatas et al., 2013). It was also found that Hox and Meis1 expression levels could be decreased via the inhibition of MLL1-WDR5 protein-protein interaction (Karatas et al., 2013; Kempinska et al., 2018). The endogenous expression level of Meis1 determines the severity of MLL (Wong et al., 2007). In cases with an inactivation mutation of Meis1 in fetal liver cells, myeloid transformation loses its capability for the differentiation and self-renewal of leukemia stem cells (Wong et al., 2007).

The effects and molecular mechanisms of MEIS proteins in leukemia have been well-studied (Lasa et al., 2004). In myeloid cells, MEIS1 is found in a trimeric form with PBX2 and HOXA9 (Shen et al., 1999). The trimeric complex occupies the region in which PBX2-MEIS1 DNA is bound (Shen et al., 1999). In addition, a regulatory feedback loop linked to Meis1, along with PU.1, Syk, and miR-146a, was reported (Mohr et al., 2017). Myeloid progenitor transformation with Meis1 and Hoxa9 is dependent on SYK-induced Meis1 expression (Mohr et al., 2017). PBX-MEIS1 heterodimer causes HOXA9-mediated immortality of myeloid progenitors (Schnabel et al., 2000). Intriguingly, MEIS1-PBX2 interaction provokes chemotherapy resistance towards leukemia (Schnabel et al., 2000). Cellular differentiation was induced by MEIS1 and other polycomb group proteins in AML patients (Grubach et al., 2008). The impairment of MEIS1-HOXA4/9 interaction disrupts epigenetic regulation in the chromosome; therefore, AML formation occurs (Grubach et al., 2008).

In pediatric pre-B cell acute lymphoblastic leukemia (pre-B-cell ALL) patients, the Meis1 promoter methylation level was observed as different from that of the control group (Musialik et al., 2015). Levels of Meis1 expression have also been correlated with white blood count (Musialik et al., 2015). Moreover, pre-B-cell ALL patients were observed to have E2A-PBX1 chimeric oncoprotein resulting from chromosomal translocation t(1;19), and translocation results in mutant oncoprotein (Carroll et al., 1984). E2A-PBX translocation causes inappropriate tissue-specific gene expression in pre-B cell ALL (Carroll et al., 1984; Calvo et al., 1999).

MEIS1 and MEIS2 interact with HOX and PBX variants in leukemia (Garcia-Cuellar et al., 2015). The presence of PBX3 and MEIS1 increases HOXA9-induced leukemia (Garcia-Cuellar et al., 2015). PBX3 enhances the stability of MEIS1 and PBX3-MEIS1 heterodimer triggers the further transcription of Meis1 (Garcia-Cuellar et al., 2015). The trimeric structure of HOXA9/MEIS1/PBX3 supports HOXA9-driven leukemia (Garcia-Cuellar et al., 2015). In addition, Meis2 is overexpressed in myeloid leukemia mice, resulting in the conclusion that MEIS1 and MEIS2 may have a parallel function in myeloid leukemia (Rieckhof et al., 1997; Fujino et al., 2001).

MEIS1 has been found to be a very important regulator for the differentiation of hPSCs into functional hematopoietic cells (Wang et al., 2018b). MEIS1 controls hematopoietic differentiation via targeting T-cell acute lymphocytic leukemia 1 (TAL1) and Friend leukemia integration 1 (FLI1) transcription factors (Wang et al., 2018a). MEIS2 regulates early hematopoietic differentiation in human embryonic stem cells (Wang et al., 2018a). The deletion of Meis2 differentially modulates TAL1, and thereby impairs endothelial specification and endothelial to hematopoietic transition (Wang et al., 2018b).

Aberrant expression of Tlx1/Hox11 also results in T-cell leukemogenesis via Meis1 and Meis2 (Milech et al., 2010). Meis1 or Meis2 and Tlx1 are coexpressed and interact with each other (Milech et al., 2010). Intriguingly, Tlx1 expression has not been observed in B-lineage ALL or primary lymphocytes (Milech et al., 2010). It has been found that Meis1/Hoxa9 deregulation has an important function in the progression of leukemia (Collins and Hess, 2016). Meis1 mutations lead blood cells to develop the symptoms of leukemia, as well as gain the chemoresistance and proliferation of leukemia cells by triggering other MEIS-cofactors (summarized in Table 1).

**Table:** MEIS proteins and associated factors involved in leukemia/lymphoma.

Associated factor/event	Component complex	Associated disorder	Reference
PU1	MEIS1 and PBX3	MLL	Zhou et al. (2014)
HOXA9	MEIS1 and PBX3	Leukemogenesis	Wang et al. (2006)
AML1-ETO	MEIS1	AML	Lasa et al. (2004)
FLT3	MEIS1 and HoxA9	AML	Staffas et al. (2017)
E2A-PBX1	MEIS1 and PREP1	Myeloid immortalization	Calvo et al. (1999)
Polycomb group genes (PcG)	MEIS1, HOXA4, and HOXA9	AML	Grubach et al. (2008)
SYK, PU1, and miR-146a	MEIS1 and HOXA9	AML	Mohr et al. (2017)
TLX1/HOX11	MEIS1 and MEIS2	T-cell ALL	Milech et al. (2010)
Translocation	t(10;14)(q24;q11)- HOX11(TCL3)	T-cell leukemia	Zutter et al. (1990)
Misregulated Hox genes	MEIS1, PBX2, and PBX3	Leukemia stem cells of MLL	Sitwala et al. (2008)
Aberrant MEIS1 promoter methylation	Meis1	Pediatric B-cell ALL	Musialik et al. (2015)
Impaired (MLL1)-WDR5 interaction	MEIS1 and HOXA9	MLL	Karatas et al. (2013)
HOXA9 and Meis1 downregulation	Hoxa9 and Hoxb8	Myeloid differentiation	Fujino et al. (2001)
t(4;11)(q21;q23) translocation	MLL-AF4 fusion gene and increasing Hoxa9-MEIS1	ALL	Thomas et al. (2005)

### 2.7. Lung cancer

The ectopic expression of Meis1 has been shown to inhibit cell proliferation in nonsmall-cell lung cancer (NSCLC) (Li et al., 2014). This was in parallel with the finding that the reduction of Meis1 expression led to the proliferation of NSCLC cells and cell cycle progression (Li et al., 2014). On the other hand, MEIS1, HOXA5, and T-box 5 proteins are considered as pathogenicity markers in lung cancer adenocarcinoma (Du and Zhang, 2015). Thus, PBX2 protein, one of the heterodimer partners of MEIS1, has been shown to cause cell proliferation, metastasis, and invasion by activating TGF-β, TGF-β-SMAD3, and sonic hedgehog signaling pathways (Plowright et al., 2009; Qiu et al., 2009; Risolino et al., 2014; Pan et al., 2016; Shi et al., 2017). HOXA5 and p53 work together to play a role in suppressing lung cancer cell invasion (Chang et al., 2017). HOXA5 and p53 reduce the level of matrix metalloproteinase-2 (MMP2), thus inhibiting cell invasion (Chang et al., 2017). Various HOX proteins (i.e. HOXA1, HOXA5, HOXA5, and HOXA8) have significant roles in lung carcinogenesis (Xiao et al., 2014; Chang et al., 2017; Liu et al., 2018; Zhang et al., 2018). Cell proliferation, metastasis, cellular survival, and chemoresistance are also involved in the utilization of the Wnt/β-catenin signaling pathway in small and NSCLC (Xiao et al., 2014; Chang et al., 2017; Liu et al., 2018; Zhang et al., 2018). Furthermore, the differentiation of methylated CpG of Hoxa7 and Hoxa9 genes when compared to healthy controls is a point that needs to be examined on a molecular level (Rauch et al., 2007).

HIF proteins are involved in protecting cells against the action of ROS-dependent apoptosis (Vukovic et al., 2015). In lung cancer, the effect of HIFs has been the subject of studies because of the cancer microenvironment (Vukovic et al., 2015). HIFs are subjected to transcriptional activation via MEIS1 protein (Vukovic et al., 2015). NSCLCs have abnormal and elevated HIF-1α expression (Yang et al., 2017). Blocking of HIF-1α complexes has been shown to cause increased metastasis and angiogenesis and an increase in cancer cell proliferation (Lin et al., 2017; Yang et al., 2017). The consumption of fluorodeoxyglucose (FDG) in lung cancer cells demonstrates the relationship between HIF-1α and HIF-2α (Higashi et al., 2016). Surprisingly, however, cells that have a high expression of HIF-2α are more aggressive against radiotherapy and FDG uptake (Sun et al., 2015; Higashi et al., 2016). MEIS1 decreases cell proliferation in small-cell lung cancer (Li et al., 2014). In summary, MEIS and HOX proteins may function as a tumor suppressor in lung cancers. However, further studies including tissue samples of patients are needed to validate these data.

### 2.8. Skin cancer

In melanoma cells (namely the MJT1 cell line), MEIS1 and PBX proteins enhance the stability of the HOX-PBX-DNA trimeric complex (Aulisa et al., 2009). Disruption of this trimeric complex by designed peptide amphiphiles has been shown to slow down the growth of melanoma cells (Aulisa et al., 2009). In addition, the 2-stage skin carcinogenesis mouse model has shown that Meis1 has a protumorigenic (oncogenic) function in the development of tumor and malign transformation (Okumura et al., 2014). In the metastatic melanoma cell line, both mRNA and protein expression analysis has shown that Pbx2 expression was elevated when compared to Pbx1, Pbx3, and Pbx4 (Errico et al., 2013). PBX2 and HOXB7 form a heterodimer structure and induce miR-221 and miR-222 expression (Errico et al., 2013). PBX2/HOXB7 heterodimer, along with miR-221 and miR-222, inhibits c-Fos expression and apoptosis (Errico et al., 2013). Hoxb7, which is abnormally expressed in different melanoma cell lines, has been shown to cause aberrant cell proliferation through the induction of basic fibroblast growth factor (bFGF) (Carè et al., 1996). Moreover, it has been shown that miRNA-196a modulates the expression of target genes (cadherin-11, calponin-1, and osteopontin) by regulating Hoxc8 in melanocyte and melanoma cells (Mueller and Bosserhoff, 2011).

Pbx1 is downregulated by promyelocytic leukemia zinc-finger (PLZF) protein in melanoma cells (Shiraishi et al., 2007). Furthermore, the knockdown of Pbx1 by short interfering RNAs (siRNA) leads to the suppression of cell growth (Shiraishi et al., 2007). Moreover, PBX1 interacts with HOXB7 in melanoma cells and the downregulation of Pbx1 causes Hoxb7 and target gene downregulation, including bFGF, Ang-2, and Mmp9 (Shiraishi et al., 2007). miR-495 acts as a tumor suppressor by directly targeting and downregulating Pbx3 (Chen and Xie 2018). Briefly, miR-495 downregulates proliferation, invasion, and colony formation, and induces apoptosis in melanoma tissues and cell lines (Chen and Xie, 2018).

Promoter methylation analysis of primary cutaneous melanoma has shown that Hoxa9 was hypermethylated in the samples (Gao et al., 2013). Moreover, a detailed DNA methylation analysis of all stages of human melanoma revealed that Hoxa9 DNA hypermethylation had a function in tumor development when compared to benign samples (Wouters et al., 2017).

Taken together, Meis1 and Pbx2 may be considered to have oncogenic properties in skin tumorigenesis. They contribute to the stability of the HOX-PBX-MEIS tertiary structure in DNA; thus, they trigger the proliferation of melanoma cells.

### 2.9. Oral cancer

Studies have suggested that HOX-PBX interaction allows the survival of oral malignant and squamous carcinoma cells (Platais et al., 2018). Following the disruption of HOX-PBX interaction with double active peptide, c-Fos expression increases and apoptosis takes place (Platais et al., 2018). PBX1 and hematopoietic PBX interacting proteins (HPIP) cause cell differentiation, proliferation, metastasis, and invasion in oral cancer (Okada et al., 2015). The misregulated expression of Hoxc6 and Hoxa10 cluster proteins are involved in the proliferation, survival and migration of oral cell carcinoma. Disruption of Hoxc6 and Hoxa10 gene expression could enhance tumor progression (Carrera et al., 2015; Tang et al., 2019).

One of the most prevalent cancers of the oral cavity is SCC. Expression pattern evaluation of Pbx2 (both mRNA and protein) in patients with gingival SCC (GSCC) revealed that Pbx2 was upregulated and could be a useful prognostic marker in these patients (Qiu et al., 2012). The overexpression of Pbx2 is associated with tumor size, stage, and metastasis, as well as a high expression of the valosin-containing protein (VCP) in GSCC cells (Qiu et al., 2012).

The hypermethylation of Meis1 has been identified in adenoid cystic carcinoma samples using the methylated CpG island amplification and microarray methods, but further validation studies are needed (Bell et al., 2011). On the other hand, an analysis of the global methylation status of 24 Hox genes in oral SCC (OSCC) cell lines revealed that 12 genes, including Hoxa9, were hypermethylated (Xavier et al., 2014). Hoxa9 hypermethylation was also reported in the promoter methylation analysis of OSCC patient tissues (Guerrero-Preston et al., 2011) and salivary rinses (Schussel et al., 2013), which leads to the growth advantage of the tumor, and an increase in metastasis (Uchida et al., 2014). Contrary to these findings, it was also reported that Hoxa9 was overexpressed in OSCC tissue samples (Wang et al., 2017a). Moreover, miR-139-5p inhibits cell proliferation, invasion, and migration by directly targeting Hoxa9 expression (Wang et al., 2017b). These findings indicate that further investigation is needed with large patient cohorts to make a clear statement about the function of Hoxa9 in OSCC development. Pbx2 and hypermethylation of Meis1 and HoxA9, however, may be considered prognostic markers of oral cancer.

### 2.10. Gynecologic cancers

Gynecologic cancer arises from any part of the reproductive organs of a woman. They have been categorized into 6 main types, comprising ovarian, cervical, uterine (endometrial-uterine sarcoma), vulvar, vaginal, and fallopian tube cancers (Stewart et al., 2013). Each of these types of gynecologic cancers has its own unique risk factors, development, and treatment strategies (Stewart et al., 2013). Uterine and ovarian cancers are among the most common gynecologic cancers (Stewart et al., 2013).

Ovarian cancer studies have shown that Meis1, Meis2, nuclear, and total cytoplasmic Pbx1–4 RNA expressions, and protein contents are higher than normal tissue in both nuclear and cytoplasmic locations (Crijns et al., 2007). Moreover, MEIS1 has been shown to play a key role in the early stages of ovarian cancer through its involvement and regulation of T-cell chemo-attraction (Karapetsas et al., 2018). Studies about MEIS-PBX proteins and their correlation with HPIP have suggested their potential to become key uterine cancer biomarkers. High Hpip expression correlates with the histological grade, lymph node metastasis, and relapse of cancer. It also reduces overall survival in uterine cancer (Yang et al., 2016). Similarly, lncRNA HOTAIR transcribed from the Hoxc locus of DNA in uterine cancers leads to cellular proliferation, metastasis, and radiotherapy resistance via the MAPK signaling pathway (Li et al., 2018). In addition, HOTAIR adversely affects tumor relapse and overall survival time (Li et al., 2015a; Li et al., 2015b).

An analysis of Pbx1 expression in ovarian tumor samples indicated that Pbx1 was upregulated in these samples, and the overexpression of Pbx1 led to platinum-based chemotherapy resistance in patients with ovarian cancer (Jung et al., 2016). In addition, Meox1 was overexpressed in ovarian cancer cells along with Pbx1, and the silencing effects of Pbx1 were reversed by Meox1 expression in the cell lines (Thiaville et al., 2012). It was also reported that notch receptor 3 (NOTCH3) is a transcriptional activator of Pbx1 in ovarian cancer (Park et al., 2008).

The overexpression of NOTCH3 is correlated with the overexpression of Jagged-1 and Pbx1b in cervical SCCs, yet there is no established relationship between Pbx1b overexpression and patient survival (Yeasmin et al., 2010). PBX3 is overexpressed in the cytoplasm of cervical cancer cells and tissues and its expression is related to poor prognosis, tumor diameter, pathological grade, clinical stage lymph node metastasis, invasion depth, and vascular invasion (Li et al., 2017). The function of PBX3 is likely controlled by the AKT signaling pathway in cervical cancer (Li et al., 2017). The miR-526b miRNA was reduced in various cancers, including cervical cancer and miR-526b expression leads to a decrease in Pbx3 expression in cervical cancer cells when compared to healthy cells (Li et al., 2019a). Moreover, the PBX3-mediated epithelial-to-mesenchymal transition (EMT) process is associated with invasion and metastasis that is inhibited by miR-526b in cervical cancer cells (Li et al., 2019a).

Gene expression analysis revealed that Pbx1–2 and Hoxb1 regulate Col5a2 expression, which possibly has a function in the focal adhesion pathway in endometrial cancer (Zhu et al., 2012). The benign and rare smooth muscle tumor called retroperitoneal leiomyoma is mostly found only in women and carries histopathological features that are very similar to uterine leiomyomas (Panagopoulos et al., 2015). In a case with retroperitoneal leiomyoma, it was reported that tumor cells had a t(9;22)(q33;q12) translocation, which resulted in a fusion of Ewsr1 and Pbx3 genes (Panagopoulos et al., 2015). Tumor-specific methylation analysis of HoxA9 in patients with ovarian cancer in phase II clinical trials with bevacizumab and tocotrienol chemotherapy revealed that HoxA9 methylation was increased after 1 cycle of chemotherapy (Thomsen et al., 2019). This suggested that Hoxa9 could be a possible biomarker for early response to inefficient chemotherapy in ovarian cancer patients (Thomsen et al., 2019).

miR-196b, whose expression is upregulated in recurrent epithelial ovarian cancer (EOC), leads to invasion of the ovarian cancer cells (Chong et al., 2017). Hoxa9 is downregulated by the binding of miR-196b to 3′-UTR; therefore, it could be a possible candidate for antimiR196b effect (Chong et al., 2017). The aberrantly expressed Hoxa9 in patients with epithelial ovarian cancer has no significant predictive value during first-line platinum-taxane chemotherapy (Pontikakis et al., 2017). On the other hand, a series of studies on ovarian cancer cells indicated that adaptation to the peritoneal environment and guidance of different types of stromal cells to reinforce tumor growth were stimulated by Hoxa9 (Ko and Naora, 2014). Although, the methylation analyses of Hoxa9 with large cohorts has shown that Hoxa9 is hypermethylated in both high-grade serous ovarian cancer (Montavon et al., 2012) and primary ovarian cancer patients, (Wu et al., 2007) the association of hypermethylation with the stage, histological types, grade, and ascites could not be established (Xing et al., 2015). Interestingly, a study on ovarian cancer cell line and normal tissue showed that DNA methylation of some of the analyzed genes, including Hoxa9, Hoxa10, MiR-34b, Prom1, Cables1, Sparc, and Rsk4, had an inverse correlation with their expression level (Niskakoski et al., 2014). It has been stated that Rsk4, Sparc, and Hoxa9 function as oncogenes in ovarian cancer development, since they have less promotor methylation correlated with a high-grade tumor (Niskakoski et al., 2014). The high expression of Hoxa9 in EOC patients induces P-cadherin via Cdh3 gene activation, which in turn leads to intraperitoneal dissemination of EOC (Ko and Naora 2014). The overexpression of HOXA9 is associated with poor outcomes in EOC patients and Hoxa9 regulates the activation of cancer-associated fibroblasts by TGF-β2 in EOC cells (Ko et al., 2012).

The expression of Hoxa9 is required for normal cervical physiology (Lopez et al., 2006) and some cervical cancer cells have shown a downregulation of Hoxa9 expression, which is modulated by both methylation and HPV infection (Alvarado-Ruiz et al., 2016). Moreover, targeting the human oncogene B-cell-specific moloney murine leukemia virus integration site 1 in the cervical cancer cell line leads to Hoxa9 activation (Chen et al., 2011). On the other hand, another study showed that Hoxa9 was expressed in both cervical carcinoma cell lines and normal cervical tissues (Hung et al., 2003).

Global methylation analysis of Hoxa9 expression in endometrial cancer tissues and healthy tissues revealed that Hoxa9 was hypermethylated in cancer tissues (Chen et al., 2015) and it was associated with lymphovascular tumor invasion (Makabe et al., 2019). Moreover, hypermethylation of Hoxa9 was also shown in DNA samples isolated from the vaginal tampon of endometrial cancer patients (Bakkum-Gamez et al., 2015).

In summary, studies have suggested that MEIS and PBX proteins may function as oncogenes in gynecologic cancers. Interactions between MEIS1 and PBX1–3 are involved in the early stages of ovarian cancer. In addition, they are highly expressed in gynecologic cancers when compared to healthy tissue. However, when all of the results are evaluated, the oncogenic or tumor-suppressive role of Hoxa9 in gynecologic cancers is inconclusive.

### 2.11. Pancreatic cancer

MEIS proteins and their partner PBX1 have been shown to cooccupy a promoter of keratin 19 during pancreas development (Deramaudt et al., 2006). T3M4, a cellosaurus cell line, is known to have stimulated cell proliferation through the formation of HOXB2-A10-PBX HD heterodimers and associated pancreatic carcinogenesis (Aulisa et al., 2009). When HOXB2-A10-PBX HD complex and target DNA interaction is impaired with a specifically designed peptide, cell proliferation significantly decreases (Aulisa et al., 2009). MEIS1 is an important transcription factor that regulates the expression of mitochondrial genes (Tomoeda et al., 2011). MEIS1 protein may alter mitochondrial activity and it is associated with metabolic pathways in pancreatic cancer (PaC) (Tomoeda et al., 2011). Studies have shown that the expression of mitochondrial genes could be downregulated when Meis1 specific siRNA was transfected into PaC cells (Tomoeda et al., 2011). In addition, MEIS proteins could contribute to the Warburg effect to facilitate the abnormal growth of cells in the hypoxic tumor microenvironment via transactivation of HIFs and cooperation with HOX proteins. HOX proteins are also known to help the survival of cancer cells by activating ERK1/2 signaling pathways and modulating epigenetic pathways in cancer cells (Tsuboi et al., 2017). Meis1 is overexpressed in primary pancreatic ductal adenocarcinoma cells and results in activation of the melanoma cell adhesion molecule that causes cell migration (von Burstin et al., 2017). On the other hand, global gene expression analysis of nonmetastatic pancreatic endocrine neoplasms and metastatic pancreatic endocrine neoplasms revealed that Meis2 was downregulated in metastatic pancreatic endocrine neoplasms (Hansel et al., 2004).

Pbx1 was differentially expressed in inflammation-associated pancreatic stellate cells (PaSCs) when compared to tumor-associated PaSCs (Scarlett et al., 2011). Mesothelin is upregulated in most PaCs and causes miR-198 downregulation via NF-κB-mediated OCT-2 induction (Marin-Muller et al., 2013). In addition, the inhibition of miR-198 leads to the upregulation of Pbx-1 and VCP, which causes tumor progression (Marin-Muller et al., 2013). In PaC cells, overexpression of Pbx3 is associated with tumor development and miR-129-5p prevents the proliferation and migration of cancer cells by targeting Pbx3 (Qiu et al., 2019).

The lncRNA HOXA transcript at the distal tip (HOTTIP) is upregulated in pancreatic CSCs (PCSCs) and pancreatic cell lines (Fu et al., 2017). HOTTIP is an important factor for the maintenance of PCSCs (Fu et al., 2017). Binding of the HOTTIP to WDR5 induces Hoxa9 expression, which is also a significant factor for PCSCs maintenance (Cheng et al., 2015; Fu et al., 2017). The mir-210, a hypoxia-inducible miRNA, inhibits tumor growth of PaC by targeting Hoxa1, Fgfrl1, and Hoxa9 (Huang et al., 2009).

In summary, MEIS, PBX, and HOX proteins may function as oncogenes in PCs. MEIS1 and PBX3 provide metastatic capabilities to pancreatic tumors. PBX1 is involved in tumor progression. However, MEIS2 demonstrates low expression in metastatic PaCs (Hansel et al., 2004).

### 2.12. Prostate cancer

Mutations in Hoxb13 generate a critical risk of developing prostate cancer (PC) (Johng et al., 2019). HOXB13, which enables the coactivation of the androgen receptor (AR) and Foxa1, forms a heterodimer with MEIS1 (Johng et al., 2019). Expression levels of butyrylcholinesterase and TNF superfamily member 10 were decreased in PC cells by MEIS1 (Decker and Ostrander 2014; Johng et al., 2019). MEIS proteins have been shown to be involved in the progression of PC by modulating the c-MYC signaling pathway, cellular proliferation, and were associated with the invasiveness of PC (Bhanvadia et al., 2018). Depletion of Meis1 and Meis2 in vivo may cause tumor growth and an increase in the expression of protumorigenic genes c-Myc and CD142 (Bhanvadia et al., 2018). Thus, the expression of Meis1 and Meis2 is related to the inhibition of metastasis in PC (Bhanvadia et al., 2018). Moreover, a MEIS cofactor, PBX3, is considered as an important biomarker in aggressive PC (Ramberg et al., 2016).

The androgen and AR have important functions during the development and maintenance of PC (Cui et al., 2014). The potential link between MEIS1 and AR has been investigated in PC cell lines. In the presence of synthetic androgen, MEIS1 prevents the transcriptional function of AR by controlling the translocation of AR from the cytoplasm to the nucleus. MEIS1 also inhibits AR binding to the prostate-specific antigen gene promoter and enhancer regions (Cui et al., 2014). In addition, MEIS1 suppresses the proliferation and anchor-independent growth of PC cells (Cui et al., 2014). The hypermethylation and transcriptional downregulation of Meis2 was shown by DNA methylation and RNA expression analyses of PC tissue samples (Norgaard et al., 2019).

Castration-resistant PC (CRPC) has a poor prognosis with a challenging treatment (Jeong et al., 2017). Progression of androgen-sensitive PC to CRPC occurs via activation of an inflammatory signaling pathway consisting of IκBα/NF-κB(p65), MEIS2, miR-196b-3p, and PPP3CC (calcineurin catalytic subunit γ isoform) (Jeong et al., 2017). MEIS2 regulates PPP3CC-directed suppression of IκBα/p65/miR-196b-3p pathway, and therefore inhibits the development of CRPC (Jeong et al., 2017). Gene array analyses of seminal vesicle epithelial cells and normal human prostate epithelium cultures (PrEC) reported that 15 Hox genes (Hoxa13, Hoxb2-3, Hoxb5-9, Hoxb13, Hoxc6, Hoxd1, Hoxd3-4, and Hoxd10-11), and their cofactors Meis1 and Meis2, were differentially expressed in PrECs (Chen et al., 2012). During embryogenesis, TWIST1 and HOXA9 function together for organogenesis of the prostate and they are overexpressed in PC cells (Malek et al., 2017). The overexpression of Twist1 leads to migration, invasion, resistance to anoikis, and metastasis in PC cells. Hoxa9 expression is regulated by TWIST1 (Malek et al., 2017). Additionally, the inhibition of Hoxa9 is enough to abolish TWIST1-promoted metastasis in PC (Gajula et al., 2013). Moreover, while Meis1, Meis2, and Pbx1 expressions are downregulated, Hoxa9 expression is upregulated during the tumor initiation and progression of PC (Chen et al., 2012). Therefore, these factors could be useful for both diagnostic and therapeutic purposes of PC (Chen et al., 2012).

Benign prostate tissue expresses PBX1 and PBX3 in the nucleus of the basal cells (Ramberg et al., 2016). On the other hand, immunohistochemical staining has revealed that cells are PBX3-positive and PBX1-negative in malign prostate tissue (Ramberg et al., 2016). Moreover, PBX3 localization shifts from the nucleus to the cytosol in malign tissue (Ramberg et al., 2016). Furthermore, multiple PBX3 isoforms have been reported in PC (Ramberg et al., 2016). Interestingly, patients who present with moderate PBX3 staining in their PC cells develop CRPC earlier than patients with strong staining (Ramberg et al., 2016). Pbx1 is downregulated in an androgen-independent PC cell line that overexpresses the Plzf, which is an androgen-responsive gene (Kikugawa et al., 2006). Moreover, Pbx1 and Hoxc8 expression cause androgen-independent growth in PC cell lines (Kikugawa et al., 2006). Interestingly, Pbx1 overexpression has also been shown in PC (Liu et al., 2019). Additionally, PBX1 leads to both cell proliferation and chemoresistance in PC (Liu et al., 2019). Moreover, the stability of PBX1 is controlled by a deubiquitinase USP9x, which decreases the polyubiquitination level of PBX1 (Liu et al., 2019). Furthermore, targeting USP9x in Pbx1-expressing PC cells leads to apoptosis (Liu et al., 2019). In PC cells, the expression level of Pbx3 is upregulated. This is achieved posttranscriptionally by Let-7d, which is an androgen-regulated microRNA that is downregulated in PC (Ramberg et al., 2011).

In PC, the high expression of Meis1 and Meis2 is required for the growth of the tumor (Bhanvadia et al., 2018). In addition, the expression of Meis1/2 is associated with antimetastasis in PC (Bhanvadia et al., 2018). Thus, Meis1/2 expression determines the aggressiveness of PC. Pbx1–3 and Hoxa9, on the other hand, may function as oncogenes in PC progression.

### 2.13. Sarcoma

Mesenchymal cells that are able to differentiate various tissues, including adipose, muscle, fibrous, cartilage, and bone, could give rise to sarcoma (Dancsok et al., 2017). Therefore, sarcoma is really a complex disease and thus far, more than 70 types of sarcoma have been described (Dancsok et al., 2017). Sarcoma is genetically complicated as well, such that an increase in mutational burden, complex karyotype, translocation, and amplification could be the genetic basis of the disease (Dancsok et al., 2017). Ewing sarcoma is one of the most prevalent and overwhelming primary bone cancers that has been seen both in children and adolescents (Lin et al., 2019). It has been shown that MEIS1 is an important factor for cell proliferation in Ewing sarcoma cells (Lin et al., 2019). In addition, in vivo silencing of Meis1 remarkably suppresses xenograft tumor growth (Lin et al., 2019). Moreover, it was reported that MEIS1 functions together with EWS-FLI1 in Ewing sarcoma (Lin et al., 2019). G-protein coupled receptor 64 (GPR64) expression is increased in Ewing sarcoma (Richter et al., 2013). Moreover, the downregulation of Gpr64 leads to the downregulation of tumor growth and metastasis, as well as the upregulation of Pbx2, Slit2, and Mbd2 (Richter et al., 2013).

Meis1-Ncoa2 fusion transcript has been reported in 2 cases with primary renal sarcoma (Argani et al., 2018). The vascular invasion, cellular necrosis, and perinephric fat invasion seen in both cases indicated that the Meis1-Ncoa2 fusion gene could be malignant (Argani et al., 2018). The aggressive soft tissue sarcoma of malignant peripheral nerve sheath tumors (MPNSTs) can occur both sporadically and together with neurofibromatosis type 1 (Patel et al., 2016). RNAi screening in MPNST cells revealed that Meis1 is an important factor (functioning as a potent oncogene) for MPNST tumor development (Patel et al., 2016). It was reported that proliferation and cell survival of MPNSTs by MEIS1 occur via P27Kip inhibition (Patel et al., 2016). The expression of miR-873 is decreased in osteosarcoma (OS) tissues and cell lines (Liu et al., 2019). The downregulation of miR-873 is related with tumor size, clinical stage, and distant metastasis (Liu et al., 2019). On the other hand, Hoxa9 expression is upregulated in OS tissues, and the silencing of Hoxa9 recovers the miR-873 downregulation effects. This indicates that Hoxa9 is the target of miR-873 in OS (Liu et al., 2019).

Taken together, Meis1 and Hoxa9 may function as an oncogene in sarcoma (Lin et al., 2019). The silencing of Meis1 in the xenograft model was found to suppress tumor size in sarcoma (Lin et al., 2019). Tumor suppressor roles of PBX2 have also been demonstrated in sarcoma (Richter et al., 2013).

### 2.14. Thyroid cancer

Vriens et al. (2011) showed that Meis2 expression in thyroid cancer patients older than 40 was about 2.5 times more frequent (Vriens et al., 2011). Moreover, gene ontology analysis of 100 genes in 4 classes of thyroid carcinoma, including papillary thyroid carcinomas, oncocytic variants of follicular thyroid tumors, tumors of uncertain malignant potential, and follicular adenomas, revealed that Hoxa9 is 1 of the 7 genes that is differentially expressed in thyroid carcinomas and is responsible for tumor development (Jacques et al., 2013).

A comparison of gene expression in MSA, K-119, KOA-2, 8305C, TCO1 anaplastic thyroid cancer cell lines, and normal thyroid tissue revealed that the expression of Hox genes maybe a predictive marker in thyroid cancer (Takahashi et al., 2004). While Hoxd9 is expressed only in normal healthy tissue, Hoxb4 is expressed in anaplastic thyroid cancer cells (Takahashi et al., 2004). Hoxb1, Hoxd10, Hoxc12, and Hoxd13 were not detected in normal thyroid tissue or anaplastic thyroid cancer (Takahashi et al., 2004). In a different study, Hoxa13, Hoxb13, Hoxc13, and Hoxd13 genes were shown to have abnormal expression in adenoma, papillary, and follicular thyroid cancer tissues, and this was confirmed at the protein level (Cantile et al., 2013). Hoxc10 triggers metastasis and invasion in thyroid cancer and the expression of Hoxc10 is inversely related to patient survival time (Feng et al., 2015).

In addition, HOTAIR transcribed from Hox locus has a significant effect on various thyroid cancers (Wang et al., 2019a). HOTAIR is upregulated about 80 times in thyroid cancer (Wang et al., 2019a) and promotes metastasis of thyroid carcinoma cells (Zhang et al., 2017). When HOTAIR is downregulated, cell proliferation is inhibited (Zhang et al., 2017).

Although it still requires further confirmation, Meis2 and Hoxa9 appear to have oncogenic features in thyroid tumorigenesis. In addition, various Hox isoforms and HOTAIR also have high expression in thyroid cancer cells and tissues. However, there is no generalization for all Hox isoforms. Some Hox isoforms also have tumor-suppressing properties.

### 2.15. Thymoma

Thymoma is among the most frequently seen subtype of thymic tumors (Comacchio et al., 2019). It is a tumor of mediastinum that originates from the thymic epithelium (Comacchio et al., 2019). It can occur at any age with the same incidence in both men and women (Comacchio et al., 2019). The upregulation of Meis1 has been observed in mice thymic epithelial cells (TECs) after the induction of thymic atrophy by dexamethasone (Popa et al., 2007). In addition, it was also shown that Meis1 is essential for the postnatal thymic microenvironment in TECs (Hirayama et al., 2014). Moreover, an in silico gene expression analysis of medullary TECs showed that Meis1 and Hoxa7 are upregulated (Rattay et al., 2016). It was reported that PBX2 has important functions in T-cell development at both the postnatal and embryonic stages of mice (Penkov et al., 2005).

**Figure 1:**
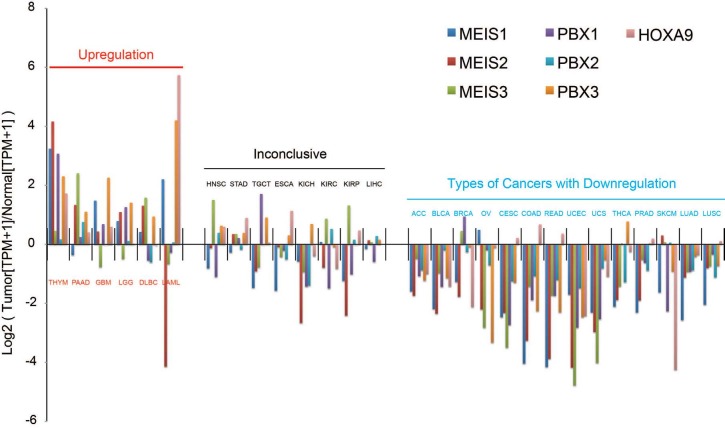
Expression profile of Meis1-3, Pbx1-3, and a commonly associated Hoxa9 gene in cancer tissues when compared to adult healthy tissue. Cancer types could be separated into 3 groups based on the overall expression of Meis and its associated factors: upregulated, inconclusive, and downregulated groups of cancers. Note that these are the average of all of the subtypes in the indicated cancers. ACC: adrenocortical carcinoma, BLCA: bladder urothelial carcinoma, BRCA: breast invasive carcinoma, CESC: cervical squamous cell carcinoma and endocervical adenocarcinoma COAD: colon adenocarcinoma, DLBC: lymphoid neoplasm diffuse large B-cell lymphoma, ESCA: esophageal carcinoma, GBM: glioblastoma multiforme, HNSC: head and neck squamous cell carcinoma, KICH: kidney chromophobe, KIRC: kidney renal clear cell carcinoma, KIRP: kidney renal papillary cell carcinoma, LAML: acute myeloid leukemia, LGG: brain lower grade glioma, LIHC: liver hepatocellular carcinoma, LUAD: lung adenocarcinoma, LUSC: lung squamous cell carcinoma, OV: ovarian serous cystadenocarcinoma, PAAD: pancreatic adenocarcinoma, PRAD: prostate adenocarcinoma, READ: rectum adenocarcinoma, SKCM: skin cutaneous melanoma, STAD: stomach adenocarcinoma, TGCT: testicular germ cell tumors, THCA: thyroid carcinoma, THYM: thymoma, UCEC: uterine corpus endometrial carcinoma, UCS: uterine carcinosarcoma.

Publications about thymoma and the analysis of Meis1-3, Pbx1-3, or Hoxa9 expression in thymoma are limited. Jia et al. (2018) showed that expressions of Meis1-3, Pbx1-3, and Hoxa9 genes were higher than those in normal tissue. In addition, previously published gene array data (Jia et al., 2018) were analyzed and differentially expressed genes were determined in various cancers, including thymoma, in comparison with healthy tissue (Figure 1). This analysis revealed that Meis1-3, Pbx1-3, and Hoxa9 genes are highly upregulated in thymoma when compared to healthy adult tissues. Further studies involving thymoma cell lines, ex vivo tumor samples, and in vivo mouse models are still necessary to determine the relationship of these genes with the development of thymoma.

### 2.16. Comparison of MEIS expression in various cancer types and their corresponding health tissues

The role of MEIS proteins have been largely studied in embryonic development and cancer progression; however, their function in adult tissues have still not been fully established (Toresson et al., 2000; Choe et al., 2002; Zhang et al., 2002; Zhang et al., 2006). Several adult tissues demonstrate high levels of Meis expression (Uhlen et al., 2015). Meis1, Meis2, and Meis3 are highly expressed in female reproductive tissues (Uhlen et al., 2015; Wu et al., 2016). MEIS1 organizes sex steroid hormones and reproduction with HOXA10 and PBX2 interactions (Sarno et al., 2005; Hu et al., 2014). Adrenal glands have a high expression of Meis1, followed by the adrenal cortex, colon, appendix, and ovaries (Wu et al., 2016). Unlike Meis1, the expression of Meis2 is elevated in the brain, pancreas, and prostate (Wu et al., 2016). MEIS2, which regulates neurogenesis with cofactors such as PAX6, FOXP1, FOXP2, FOXP3, and PBX3, provides dopaminergic periglomerular fate specification (Co et al., 2020; Agoston et al., 2014; Grebbin et al., 2016). MEIS3, which is overexpressed in the brain, interacts together with PBX4, HOXB1B, and manages the hindbrain fate utilization of the FGF/MAPK and planar cell polarity signaling pathway (Aamar and Frank, 2004). MEIS3 in the hindbrain has a regulatory role that induces neural progenitor cells and maintains the neural stem cell pool (Vlachakis et al., 2001; Waskiewicz et al., 2001).

According to the GEPIA and BioGPS datasets, the expression of Meis1, Meis2, and Meis3 vary in different cancers (Wu et al., 2013; Wu et al., 2016; Tang et al., 2017). Interestingly, Meis1 is overexpressed in lymphoid neoplasm diffuse large B-cell lymphoma, cholangiocarcinoma, kidney renal clear cell carcinoma, ovarian serous cystadenocarcinoma, glioblastoma multiforme, acute myeloid leukemia, brain lower grade glioma, and thymoma (Wu et al., 2013; Wu et al., 2016; Tang et al., 2017). The Meis2 expression pattern in cancer tissues differs from that of Meis1, and it also differs in adult tissues. Meis2 has a high expression profile in cholangiocarcinoma, glioblastoma, brain lower grade glioma, pancreatic adenocarcinoma, liver hepatocellular carcinoma, skin cutaneous melanoma, stomach adenocarcinoma, and thymoma. MEIS3, which is the least studied isoform of the MEIS proteins, is highly expressed in breast-invasive carcinoma, diffuse large B-cell lymphoma, kidney renal papillary cell carcinoma, head, and neck SCC, and pancreatic adenocarcinoma, unlike other isoforms (Wu et al., 2013; Wu et al., 2016; Tang et al., 2017). It is not possible to identify a cancer in which all of the MEIS isoforms are expressed at a high level. As in normal tissues, the expressions of MEIS in cancers are quite different from each other. Only in lower-grade brain glioma are the expressions of all MEIS isoforms higher when compared to healthy tissue (Wu et al., 2013; Wu et al., 2016; Tang et al., 2017).

A study by Jia et al. (2018) demonstrated the profiling of TALE and HOX protein members in cancers and revealed a complex network of MEIS isoforms in cancer biology. The expression of MEIS isoforms varied in almost all cancer tissues when 28 different cancer types were studied. Meis1, Meis2, and Meis3 were upregulated in lymphoid neoplasm diffuse large B-cell lymphoma and thymoma cancers. Meis1 was upregulated in acute myeloid leukemia and glioblastoma. In other cancers, the expression of Meis1 was either reduced or did not lead to a significant change. Meis2 and Meis3 expression were increased in pancreatic adenocarcinoma. Meis3, unlike other isoforms, was increased in head and neck SCC (Jia et al., 2018).

Overall, the expression of Meis1–3, Pbx1–3, and their associated partner, Hoxa9, were observed as upregulated in thymoma, pancreatic adenocarcinoma, glioblastoma, glioma, lymphoma, and leukemia when compared to corresponding healthy adult tissues (Figure 1). In particular, Meis1 was upregulated in breast, neuroblastoma, gynecologic, skin, sarcoma, and thymoma cancers, which may be associated with cancer cell survival, proliferation, and tumorigenesis. Meis1 and Meis2 were upregulated in parallel, only in neuroblastoma, gynecologic, and thymoma cancers. MEIS2 has been shown to induce metastasis in bladder cancer (Xie et al., 2019). On the other hand, MEIS1 has a tumor suppressor role in some cancers. MEIS1 has a cancer growth suppressor role in kidney and PC (Chen et al., 2012; Zhu et al., 2017). MEIS2 may also function as a tumor suppressor in PC (Chen et al., 2012). In PC, there is no tumor aggression in patients with high Meis1 and Meis2 expression (Bhanvadia et al., 2018). MEIS1 could trigger cell proliferation in colorectal cancer, while MEIS2 determines the relationship between colorectal cancer growth and death (Wan et al., 2019). MEIS2 could increase colorectal dependent cell death (Wang et al., 2019b). In glioma, Meis1 expression is high, while Meis2 expression is low. Unfortunately, studies on the role of Meis3 in cancer biology are very limited. Given the importance of MEIS isoforms and cofactors in cancer biology, it is clear that the effects of MEIS3 on cancer biology need to be investigated. In fact, the same MEIS isoform, along with associated cofactors, may have 2 different functions, as a tumor suppressor or an oncogene, in different cancers. This requires further analysis and understanding of the complex interaction of MEIS proteins in different cofactors to form a conclusion about the final outcome of MEIS activity in cancer, as well as adult tissues.

## 3. Noncoding RNA-based therapies in cancer and modulation of MEIS and its partner expression

Noncoding RNAs are involved in various biological processes, including the regulation of translation, RNA splicing, DNA regulation, gene regulation, genome defense, and chromosome structure, and may act as a hormone (Sanchez Calle et al., 2018). The expression of noncoding RNAs has often been found to impair cancer (Di Leva et al., 2014; Hayes et al., 2014; Sanchez Calle et al., 2018). Noncoding RNAs, especially siRNAs and lncRNAs, have the potential to be used as chemotherapeutic agents in cancer treatments (Drosopoulos and Linardopoulos, 2019). siRNA, which is a double-stranded RNA molecule with a length of 20–25 base pairs, is complementary to the mRNA of the specific gene (Yarian et al., 2019). Therefore, the expression level of an oncogene can be targeted by limiting the translation level with siRNA treatments (Drosopoulos and Linardopoulos, 2019; Yarian et al., 2019). Several siRNAs have been shown to reduce cancer proliferation, metastasis, invasion, and impaired cell cycle (Yarian et al., 2019). In addition, some siRNAs could stimulate apoptosis in PC, OSCC, lung cancer, nasopharyngeal carcinoma, osteosarcoma, and others (Feng et al., 2017; Liu et al., 2017; Shi et al., 2018; Wang et al., 2018c; Wang et al., 2018d; Wang et al., 2018e; Wang et al., 2019c)

miRNAs could act as tumor suppressors or oncogenes (Calin et al., 2002; Peng and Croce, 2016). For instance, miR-888 promotes the spread of cancer to the body via metastasis and invasion (Li et al., 2019b). miR-31, miR-34, miR-182, miR-211, and miR-599 expression are positively correlated with Satb2 in carcinogenesis (Chen et al., 2019). miR-206 reduces Pax3 and Met gene expression and leads to the reduction of malignancy of osteosarcoma (Zhan et al., 2019). On the other hand, miR-181c and miR-133b have the opposite effect by increasing the sensitivity of chemotherapies (Han et al., 2019). However, some studies have shown that miR-133b causes resistance to chemotherapy (Lin et al., 2018). miR-148a, miR-374b, and miR-433 decrease cell proliferation and invasion in pancreas and cervical cancer, and NSCLC, respectively (Liu et al., 2018; Sun et al., 2019; Xia et al., 2019). The outlined functions of miRNAs are new evidence to be targeted for chemotherapy (Sun et al., 2019).

lncRNAs, which are more than 200 nucleotides in length, act as negative or positive regulators in differentiation and development processes, and provide homeostasis (Carninci and Hayashizaki, 2007; St Laurent et al., 2015; Renganathan and Felley-Bosco, 2017). lncRNAs are involved in epigenetic regulation, posttranscriptional regulation could act as either a tumor suppressor or oncogene in cancer (Bach and Lee, 2018). In addition, lncRNAs can be used for the purpose of early diagnosis and prognosis of cancer (Di Leva et al., 2014; Hayes et al., 2014). lncRNA CASC11, TUG1, PCAT6, LOC730100, and LINK-A have important functions in the proliferation and metastasis of the bladder cancer, laryngocarcinoma, cervical, glioblastoma, and ovarian carcinoma (Li et al., 2019c; Luo et al., 2019; Lv et al., 2019; Zhang et al., 2019; Zhuang et al., 2019). Surprisingly, it was also observed that lncRNA MALAT1 had effects on energy metabolism that are known to be associated with metastasis in hepatocellular carcinoma and the upregulation of glycolysis genes (Malakar et al., 2019). Thus, the relationship of lncRNAs involved in the Warburg effect, which is an important phenomenon in cancer metabolism, have been shown (Malakar et al., 2019).

When mouse embryonic fibroblast cells were transfected with shPbx, the levels of MEIS1 and MEIS1-PREP1a were also reduced at the protein level (Dardaei et al., 2014). MLL-AF4 siRNA treatment also led to a reduction of the expression levels of Hoxa9 and the Meis1 signaling pathway, which in turn, prevented engraftment and clonality (Thomas et al., 2005). Moreover, Hoxa9 specific siRNA transfected THP cells demonstrated increased apoptosis within 5 days (Faber et al., 2009). When human EZH2, a member of the family of polycomb repressive complex, is suppressed by siRNA, the expression levels of Meis1 and Hoxa9 are reduced in human acute leukemia cells (Fiskus et al., 2006). In the PC cells (i.e. Panc1), mitochondrial activity is decreased by the suppression of TALE protein family members by siRNA (Tomoeda et al., 2011). Targeting of Meis and its partners by siRNA suggests an awaiting potential for an in vitro chemotherapeutic approach by modulating related mitochondrial and metabolic pathways.

## 4. Targeting MEIS, its associated partners and pathways in cancer

Small molecules alter the activity or function of target macromolecules by binding to them (Arkin and Wells, 2004). Small molecules could inhibit protein-protein/DNA-protein interactions; therefore, the utilization of small molecules allows an understanding of the biological processes and could be used in the treatment of various diseases (Arkin et al., 2014). Small molecules that are effective in cellular signaling pathways are easily diffused intracellularly, and thus can be transported very rapidly inside of the intercellular region (Yang and Hinner, 2015). Except for protein-based drugs, the majority of pharmaceutically active compounds are small molecules (Lu et al., 2011).

Over recent decades, small molecules have been intensely studied for their utilization in cancer therapy. MEIS1 has been shown to transactivate HIF-1α and HIF-2α expression, modulate cellular metabolism, and redox state (Simsek et al., 2010; Kocabas et al., 2012; Mahmoud et al., 2013; Aksoz et al., 2018). There is a direct correlation between HIF-1α and mitochondria activity in healthy cells. HIF-1α and HIF-2α protect the cell against ROS-dependent apoptosis. Some of the small molecules that disrupt mitochondria activity via inhibiting HIFs lead to cancer cell apoptosis (Polson et al., 2018; Wei et al., 2018; Marciano et al., 2019).

MEIS, associated HOX proteins, and downstream pathways may be prominent chemotherapy targets for cancer treatment. To this end, various studies over the last decade have targeted the TALE family of proteins and their interaction with MEIS proteins with small molecules or peptides in cancer. A peptide, for instance, designed to disrupt the HOX/PBX/DNA interaction reduced the proliferation of T3M4 PC cells, K562 leukemia cells, and MJT1 melanoma cells (Aulisa et al., 2009). Two small peptides, HXR9 and CXR9, were shown to induce apoptosis in NSCLC cells, breast, ovarian, prostate, and meningioma cells by disrupting the interaction of HOX and PBX (Plowright et al., 2009; Morgan et al., 2010; Morgan et al., 2012; Ando et al., 2014). In neuroblastoma, stable PBX1-MEIS1 interaction provides an overexpression of Phox2b (Di Zanni et al., 2015). PHOX2B expression was reported as reduced in neuroblastoma cells as a result of small molecule combination, which included curcumin, SAHA, and trichostatin (Di Zanni et al., 2015).

Several MLL cell lines have overexpressed Meis1 and Bcl2 (Somers et al., 2016). As a result of small-molecule library screening, it was observed that small-molecule CCI-007, although not specific for MEIS1, had a cytotoxic effect within few hours in CALM-AF10, MLL-r, and SET-NUP214 leukemia cell lines (Somers et al., 2016). Small-molecule CCI-007 triggers caspase-dependent apoptosis due to mitochondria depolarization and alters the expression of MLL-related genes (Somers et al., 2016). MLL cell lines have different metabolic phenotypes and mitochondrial respiration through different molecular pathways (Somers et al., 2019). CCI-006 also impairs mitochondria in the case of CCI-007 and increases apoptosis. Intriguingly, small-molecule CCI-006 demonstrates a cytotoxic effect in MLL, specifically in a low Meis expressing subset of cells. This suggests that MEIS+ cells may not be affected by CCI-007-induced apoptosis.

## 5. Novel small-molecule MEIS inhibitors

Studies over the last decade have shown that MEIS and its partner proteins have crucial roles in regeneration, stem cell/progenitor function, cellular metabolism, RLS, and tumorigenesis (Wang et al., 2018a; Sarayloo et al., 2019; Paul et al., 2020). Various cancers overexpress MEIS proteins and their cofactors. MEIS proteins have been found to interact with PBX1 and HOXA9 while driving tumorigenesis (Collins and Hess, 2016). Small-molecule inhibitors blocking PBX1-DNA interaction have been recently described (Wang et al., 2018f). These inhibitors disrupt DNA-protein interaction instead of protein-protein interaction; therefore, they are more efficient than previously developed inhibitors in terms of in vitro usage, cellular penetration, and/or solubility (Wang et al., 2018f).

**Figure 2:**
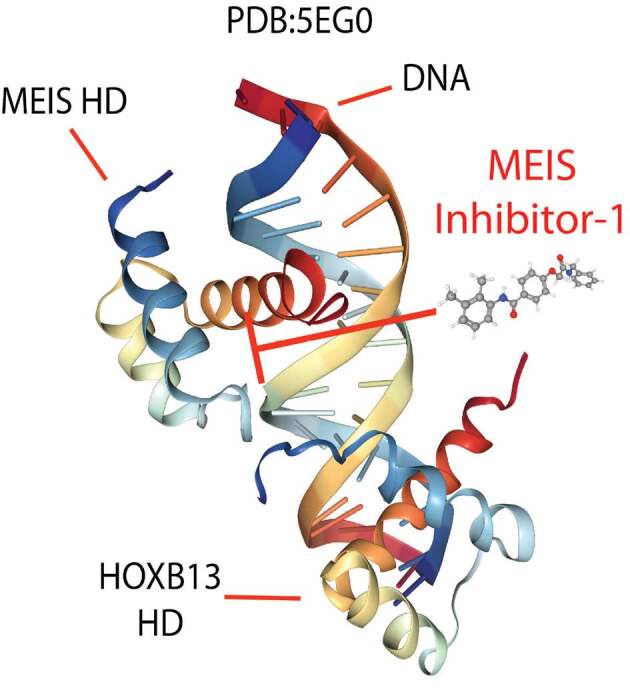
Newly developed small-molecule MEIS inhibitors target HD-DNA interaction. Crystal structure of MEIS and HOXB13 homeodomains bound to DNA are shown.

Recently, our proprietary tools and expertise were used in MEIS biology to develop MEIS inhibitors (Kocabas, 2019; Turan et al., 2020). Also performed were in silico, in vitro, ex vivo, and in vivo assays to validate small-molecule MEIS inhibitors. As a result, 2 newly identified small-molecule MEIS inhibitors (namely MEISi-1 and MEISi-2) are cell-permeable and dose-dependent, and specifically inhibit MEIS HD-DNA interaction; thereby preventing the transactivation of MEIS-targeted gene expression (Figure 2). They have a high affinity and preferential binding to MEIS HD in comparison to other TALE families of HD proteins. They inhibit MEIS-Luc activity in a dose-dependent manner as low as 100 nM. Meis1 is known to transcriptionally regulate the expression of HIF-1α, HIF-2α, and p21 (Kocabas et al., 2012; Muralidhar and Sadek, 2016). MEIS inhibitors downregulate the expression of Meis1, Meis2, and MEIS target genes, including HIF-1α, HIF-2α, and p21. They demonstrate a tissue and cell type-specific phenotype, and MEISi-1 and MEISi-2 could induce human and mouse hematopoietic stem cell expansion, self-renewal, and increased CFU-GEMM. The expression of Meis1 and HIF-2α is downregulated by them in bone marrow. They induce mouse hematopoietic stem cell expansion in vivo.

Studies conducted over past decades have suggested that MEIS and MEIS partners are crucial for cancer biology. The development of small molecules targeting MEIS or its cofactors in drug development can be a valuable asset in the chemotherapy of MEIS-positive cancers.

## 6. Conclusion

Since their discovery in 1995, MEIS proteins have been reported to have diverse roles in development, metabolism, homeostasis, regeneration, and cancer biology. MEIS isoforms (MEIS1, MEIS2, and MEIS3) accomplish developmental and physiological processes in collaboration with HOX and PBX proteins. MEIS proteins are able to alter the cell cycle and energy metabolism of hematopoietic stem cells by transactivating HIFs. It has been reported that cells undergo apoptosis as a result of impaired MEIS expression. Disruption of the MEIS-PBX interaction could also cause caspase-dependent apoptosis in a cell-dependent manner. Expressions of MEIS and associated proteins are tightly regulated during development and in adult tissues that contribute to overall homeostasis in the organism.

In cancer biology, Meis genes were first described as an oncogene in the leukemia mouse model. Over the last few decades, many studies have been conducted to understand the role of MEIS proteins in leukemia when compared to other cancer types. Over recent years, the use of clinical data has demonstrated that MEIS and varying MEIS partners play crucial roles in the development of various cancers, especially in neuroblastoma and cancers of the male and female reproductive system (Spieker et al., 2001; Uhlen et al., 2015) (summarized in Table 2). Although Meis was first described as an oncogene, a number of studies have demonstrated the tumor suppressor role as well (Song et al., 2017; Zhu et al., 2017). MEIS proteins could, for instance, suppress proliferation, migration, invasion, and metastasis of renal cell carcinoma and gastric cancers (Song et al., 2017; Zhu et al., 2017).

**Figure 3:**
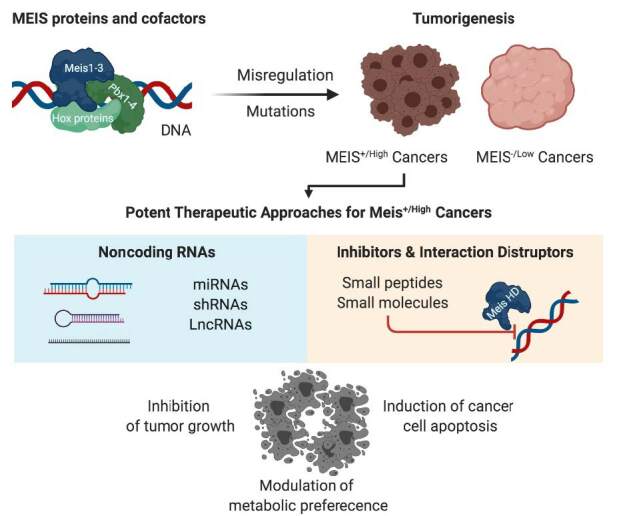
Potent therapeutic approaches for MEIS+/High cancers. Meis1-3 and its cofactors are prominent oncogenic transcription factors. Various cancers could occur due to misregulation or mutations the MEIS transcription factors, which can be classified as Meis+/High and Meis–/Low. There are 2 types of potent targeted approaches for Meis+/High cancers: RNA-based methods and inhibitors/disrupters. While RNA-based methods inhibit the translation of Meis1-3 transcripts, the disruptors bind the MEIS protein in order to block its function or interaction with other cofactors. Thus, these approaches may cause the inhibition of tumor growth, induction of cancer cell apoptosis, and modulation of metabolic preference.

Knowledge about MEIS and MEIS partner proteins is increasing gradually. The relationship of MEIS and MEIS partner proteins with various cancer types was discussed herein, as well as the treatment strategies of these cancers with small molecules or noncoding RNAs (Figure 3). While the expressions of MEIS or its partner proteins in several cancer types are increased, they are decreased in many others. Conventional chemotherapy is often painful for cancer patients and, unfortunately, the success rate is limited. Therefore, researchers aim to develop targeted chemotherapy approaches. In a number of in vitro studies where MEIS and MEIS partner proteins were targeted indirectly by small molecules or noncoding RNAs, cancer proliferation, spread, metastasis, and invasion could have been suppressed. In conclusion, MEIS and its partner proteins are key cancer biomarkers and could be therapeutically targeted in cancer treatments.

**Table 2:** Role of MEIS proteins or their cofactors in various solid cancers.

Cancer type	Involvement of the MEIS proteins or their cofactors in tumorigenesis	Reference
Bladder	MEIS2 could promote metastasis. Hoxa13 and Hoxb13 are overexpressed.	Xie et al. (2019)
Breast	Meis1 is upregulated in breast cancer	Doolan et al. (2009)
Colorectal	Meis2 expression is associated with mortality in colorectal cancer. Overexpression of Meis1 diminishes cell proliferation.	Crist et al. (2011), Wang et al. (2019)
Glioblastoma	Overexpression of Pbx3, Hoxa10-13, and Hoxc6-10 causes cell proliferation and chemotherapy resistance	Geerts et al. (2003), Jones et al. (2000), Kim et al. (2014), Manohar et al. (1993), Spieker et al. (2001)
Glioma	Meis1 is overexpressed in glioma.	Berdasco et al. (2009), Vastrad et al. (2017)
Neuroblastoma	Overexpression of Meis1 and Meis2 could induce tumor progression.	Spieker et al. (2001), Zha et al. (2014)
Kidney	Overexpression of Meis1 inhibits renal cell carcinoma cell proliferation.	Zhu et al. (2017)
Lung	Endogenous Meis1 expression could inhibit cell proliferation	Li et al. (2014)
Skin	MEIS1 promotes tumor development. PBX2 and HOXB7 determines aggressiveness in melanoma.	Errico et al. (2013), Okumura et al. (2014)
Oral	Hoxa9 is upregulated in oral cancer.	Carrera et al. (2015)
Gynecologic	Meis1-2 and Pbx1-3 are upregulated in gynecologic cancers	Li et al. (2017), Li et al. (2019), Zhu et al. (2012)
Pancreatic	Meis1 and Pbx3 give metastatic capabilities to pancreatic tumors. PBX1 is involved in tumor progression.	Qiu et al. (2019), Scarlett et al. (2011)
Prostate	Meis1 and Meis2 expression determine the aggressiveness of prostate cancer.	Bhanvadia et al. (2018)
Sarcoma	Silencing of Meis1 in a xenograft model was found to suppress tumor size	Lin et al. (2019), Patel et al. (2016)
Thyroid	Meis2 and Hoxa9 is upregulated in thyroid cancer.	Jacques et al. (2013), Vriens et al. (2011)
Thymoma	Meis1-3, Pbx1-3, and Hoxa9 are upregulated in thymoma.	Jia et al. (2018)

## 6.1. Highlights

MEIS proteins in cooperation with PBX and HOX proteins modulate tumorigenesis and development.

Meis1–3, Pbx1–3, and Hoxa9 are upregulated in thymoma, pancreatic adenocarcinoma, glioma, glioblastoma, and leukemia/lymphoma when compared to healthy tissue.

Meis1–3, Pbx1–3, and Hoxa9 are downregulated in lung, skin, and prostate cancers.

Every cancer type includes at least a subtype of cancer with MEIS+/high expressivity.
